# Radiolabeling of Micro-/Nanoplastics via In-Diffusion

**DOI:** 10.3390/nano13192687

**Published:** 2023-09-30

**Authors:** Alexandra Stricker, Stephan Hilpmann, Alexander Mansel, Karsten Franke, Stefan Schymura

**Affiliations:** 1 Helmholtz-Zentrum Dresden-Rossendorf, Institute of Resource Ecology, Research Site Leipzig, Permoserstr. 15, 04318 Leipzig, Germany; 2 Helmholtz-Zentrum Dresden-Rossendorf, Institute of Resource Ecology, Bautzner Landstraße 400, 03148 Dresden, Germany

**Keywords:** microplastics, radiolabeling, in-diffusion, Hansen Solubility Parameters

## Abstract

Micro- and nanoplastics are emerging pollutants with a concerning persistence in the environment. Research into their environmental impact requires addressing challenges related to sensitively and selectively detecting them in complex ecological media. One solution with great potential for alleviating these issues is using radiolabeling strategies. Here, we report the successful introduction of a ^64^Cu radiotracer into common microplastics, namely polyethylene, polyethylene terephthalate, polystyrene, polyamide, and polyvinylidene dichloride, which allows the sensitive detection of mere nanograms of substance. Utilizing a Hansen Solubility Parameter screening, we developed a swelling and in-diffusion process for tetraphenylporphyrin-complexed ^64^Cu, which permits one-pot labeling of polymer particles.

## 1. Introduction

Micro- and nanoplastics, i.e., plastic particles with a diameter of below 5 mm, are emerging pollutants caused by the anthropogenic use of synthetic polymer-based materials [1]. Since the 1970s, they have been found in marine environments, such as water and beaches. Today, they are common pollutants in water and soils worldwide [2,3,4]. Anthropogenic microplastic emissions originate from their intentional use in plastic products or result from the fragmentation of macroplastics during their use phase or in the environment. In Germany, microplastic emissions have been estimated at 330,000 tonnes per year or 4000 g per year per capita, with an expected increase due to rising production [5,6].

**Figure 1:**
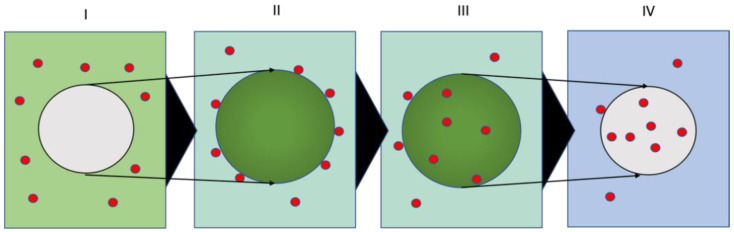
Principal steps in the labeling process of plastic particles (grey) with fluorescent dye/radio-metal carrier (red) in a water (blue)/solvent (green) mixture: (**I**) a particle in water/solvent/dye mixture, (**II**) a particle swelling with solvent and the sorption of the dyes/carriers onto the particle surface, (**III**) the diffusion of the dye/carrier molecules into the solvent-swollen polymer matrix, and (**IV**) the entrapment of dye/carrier molecules in the particles upon the removal of the solvent.

Research into the environmental fate of micro-/nanoplastics must overcome significant challenges in detecting their presence in complex ecological compartments, including water, soil, and organisms [7,8,9]. Techniques such as Raman and IR spectroscopy [10,11], mass spectrometry [12], and various spectroscopic microscopy techniques [13,14,15] are utilized for these plastics’ detection and analysis. However, the process of detecting particles often demands intensive preparation, including sampling, extraction, size separation, and purification, before analysis and quantification are viable [15,16]. Additionally, natural carbon backgrounds and artificial plastic contamination introduced during sample handling create further obstacles to their analysis [17,18]. Labeling the particles of interest for laboratory experiments is an approach that has proven useful in overcoming these setbacks. Fluorescence labeling offers a straightforward approach to detecting micro- and nanoplastics in organisms and cells [19]. This technique provides valuable insights into the potential toxic mechanisms and fates of micro- and nanoplastics in organisms [20,21]. Fluorescence-labeled plastic particles are commercially available and can be produced using appropriate staining methods [22,23,24,25]. Other labeling methods include tracking metals or nanoparticles introduced into the plastics during synthesis or substances adsorbed onto the plastic materials [26,27,28,29,30]. An additional powerful labeling technique is the use of radiotracers, which offer distinct detection and quantification possibilities [31]. For inorganic nanoparticles, radiotracers have been utilized to demonstrate and quantify particle uptake into plants, investigate the uptake mechanisms of nanoparticles into organisms, etc. [32,33]. Here, the radiotracer can be introduced easily during particle synthesis or via activation and in-diffusion techniques [34]. The radiolabel produces a radiation signal that can be readily detected outside of the sample, allowing sensitive, selective detection independent of the matrix and elemental or particle backgrounds. Excessive sample preparation is not necessary. For plastic particles, satisfactory radiotracing is difficult due to the lack of appropriate radiotracers for organic materials (the default radiotracers for organics, such as ^3^H and ^14^C, often provide little benefit over other labeling techniques, since the sample largely absorbs their low energy radiation) and more complicated organic synthesis strategies [35]. Routines exist to bind complexed radiometals to the particle surface [36]. However, this process may affect the properties of the particle surface. Here, we propose a facile in-diffusion route for radiolabeling plastics adapted from fluorescent dye labeling [37]. This technique enables the introduction of complexed radiometals into various different types of common plastic particles. Fluorescent dyes can be introduced into plastics via a swelling and in-diffusion mechanism comprising four fundamental steps (see Figure 1 I–IV) [38]. Firstly, the microplastics are brought into a mixture of water, a suitable solvent, and the dye (I). Secondly, the plastics swell based on the affinity of the solvent with the plastics, and the dye adsorbs onto the particle surface based on its affinity with the hydrophobic plastic matrix (II). Thirdly, the dye molecules diffuse into the particles governed by the sorption/desorption equilibrium and the affinity of the dye with the solvent-swollen plastic matrix, as opposed to the aqueous surrounding (III). The size of the dye molecules and the flexibility of the polymer matrix control the diffusive transport process. The matrix effects are determined by the mesh size/entanglement of the polymer strands and the glass transition temperature of the plastic particles. Finally, the dye will be trapped in the de-swollen particles after removing the solvent in a straightforward one-pot labeling routine (IV).

We investigate all of the controlling factors mentioned above. Solvent selection is guided through a Hansen Solubility Parameter (HSP) screening [39], while dye/carrier selection is guided through a hydrophobicity screening. The insights are used to achieve the radiolabeling of common micro- and nanoplastics [40] with ^64^Cu complexed by the porphyrin-derivative tetraphenylporphyrin (TPP), for the easy detection of nanograms of plastics [41].

## 2. Materials and Methods

### 2.1. Materials

An assortment of plastics was selected to represent typical micro- and nanoplastic types, including polyolefins, polyesters, and polyamides, featuring assorted structural motives (see Figure S1 for structures) [1]. The microplastics polyethylene (PE), polyethylene terephthalate (PET), polyvinylidene dichloride (PVDC), and polyamide (Nylon 12, PA) were procured from Goodfellow (Hamburg, Germany) as dry powders. Polystyrene (PS) nanoplastic particles (100 nm particle size standard) were obtained in the form of an aqueous suspension from Thermo Scientific (Bremen, Germany). The fluorescent dyes acridine orange (hydrochloride), rhodamine 6G, rhodamine B, fluorescein (disodium salt), and eosinY (disodium salt) were purchased from Magnacol Ltd. (Newtown, UK) (see Figure S2 for structures). The porphyrin derivatives TPP, copper(II), vanadate, and zinc tetraphenylporhyrins (Cu-TPP, V-TPP, Zn-TPP), along with tetrahydrofuran (THF), ethanol, 1-octanol, pluronic surfactant, and Cu(OAc)_2_, were acquired from Sigma Aldrich (Darmstadt, Germany). All chemicals were used as received. ^64^Cu was produced at the in-house cyclotron at the HZDR Research Site Leipzig [42].

### 2.2. Methods

#### 2.2.1. Characterization Methods

The particles were characterized for size, morphology, and surface texture via light microscopy using a DM-EP microscope (Leica Microsystems, Wetzlar, Germany) equipped with a Progres Gryphax camera (Jenoptik AG, Jena, Germany) and via scanning electron microscopy with energy dispersive X-ray spectroscopy (SEM-EDX) using an ITM200 SEM (JEOL, Akishima, Japan). Prior to SEM imaging, the particles received an Au/Pd (60/40) coating using a Quorum Q150TS sputter coater (Quantum-Design, Darmstadt, Germany). Size distribution data were obtained from the recorded images using the ImageJ software (version 1.53t). The PS 100 nm size standard particles were characterized via dynamic light scattering (DLS) using a Malvern Zetasizer nano (Malvern Panalytical, Malvern, UK). The UV/Vis spectra of the fluorescent dyes and TPP derivatives were recorded using a Lambda 25 UV/Vis spectrometer (Perkin Elmer, Waltham, MA, USA). The fluorescence spectra were recorded using a FluoTime 300 fluoro-meter (PicoQuant, Berlin, Germany).

#### 2.2.2. Water–Octanol Partition Coefficient Measurements

To determine the water–octanol partition coefficient *K_OW_*, 100 µL of an aqueous solution containing 2 mg/mL of dye was added to a two-phase system composed of 1.9 mL of deionized (DI) water and 2 mL of octanol. After being agitated for 15 min using a KS 260 basic orbital shaker (IKA-Werke, Staufen, Germany), the absorbance of dye in the water phase *A_W_* was measured at the peak maximum through UV/Vis spectroscopy and compared to the absorbance *A_ref_* of a 1.9 mL single-phase aqueous reference sample with the addition of 100 µL of dye solution. The water–octanol partition coefficient was then calculated as follows:

(1) $$K_{OW}=\frac{C_{O}}{C_{W}}=\frac{A_{ref}-A_{W}}{A_{W}}$$

#### 2.2.3. Swelling/De-Swelling Test

The swelling behavior of the different plastics and its reversibility was tested by dispersing 1 mg of particles (PE, PA, PVDC, PET) or 10 µL of particle dispersion (100 µg of particles, PS) in water containing 2 wt% pluronic surfactant via sonication using a Sonoplus HD3200 ultrasonic homogenizer equipped with a BR30 resonance cup (2 min, 90% amplitude; Bandelin, Berlin, Germany). A settling step of 5 min was then used to remove the largest particles of the polydisperse samples from the suspension. Size measurements were conducted in quartz cuvettes using DLS to characterize the pristine particles. Subsequently, THF was added to produce a mixture of THF and DI water with a proportion of 1:9 (by volume). DLS size measurement was performed after 5 min of treatment and 5 min of settling to observe the particle swelling. According to the literature, for the DLS measurements taken at 20 °C, the viscosity and refractive index values for the THF/water mixture were set at 1.242 mPas and 1.3377, respectively (see Figure S3) [43]. Following this step, vacuum suction was used to remove the THF, and a final DLS measurement was taken to check if the swelling was reversible. While none of the samples were monodisperse (apart from PS), this methodology facilitated reproducible z-average size measurements.

#### 2.2.4. Fluorescent Dye Labeling

**Figure 2:**
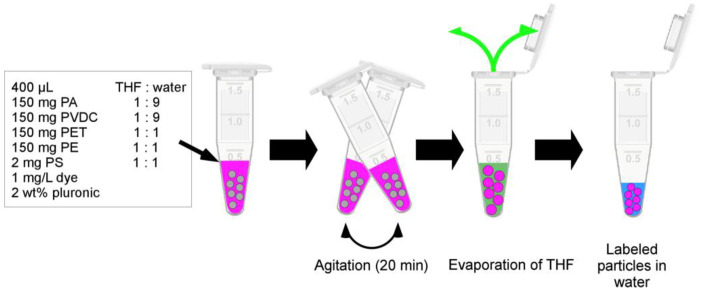
Scheme of labeling procedure. A solution of dye (pink) in THF (green) and water (blue) is added to plastic particles (grey) with the addition of surfactant as a stabilizer. During an agitation step, the particles undergo swelling, and the dye diffuses into the particles. Subsequently, the THF is evaporated, leaving the de-swollen labeled particles in an aqueous suspension.

For fluorescent dye labeling, 150 mg of particles (PE, PA, PVDC, PET) or 200 µL of particle dispersion (2 mg of particles, PS) were treated with 400 µL of mixtures composed of THF and DI water with varying THF contents, along with 1 mg/L of dye and 2 wt% pluronic surfactant. After 20 min of shaking, the samples were left open overnight to allow the THF to evaporate (see Figure 2). After this process, DI water was added to the samples to achieve a volume of one milliliter. The samples underwent sonication for 2 min and were centrifuged using an Eppendorf MiniSpin^®^ centrifuge (15 min, 14,100× *g*; Eppendorf SE, Hamburg, Germany). Subsequently, the dye concentration in the supernatant was measured using UV/Vis spectroscopy to determine the labeling yield via comparison with a reference sample. Four rounds of washing using fresh DI water were performed in a similar manner. Successful fluorescence labeling was confirmed through the use of an Olympus BX-61 fluorescence microscope (Olympus, Hamburg, Germany).

#### 2.2.5. ^64^Cu Production

^64^Cu was generated via the proton activation of a ^64^Ni enriched Ni-layer produced via electrodeposition at the in-house cyclotron [42,44]. The Ni was deposited onto a gold disc from a 0.22 M (NH_4_)_2_SO_4_ solution at pH 9, using a voltage of 2.60 V and a current of 25 mA [45]. The target was covered with a 100 µm thick aluminum foil and irradiated with 12 MeV protons for ~80 μAh using the Leipzig cyclotron Cyclone^®^ 18/9 equipped with a COSTIS^®^ solid target system (IBA Molecular, Louvain-la-Neuve, Belgium) to induce the ^64^Ni(p,n)^64^Cu nuclear reaction. The ^64^Cu was isolated from the target material via the dissolution of the target in 12.5 M HCl. This mixture was then evaporated until dry and re-dissolved in 6 M HCl. Ion exchange chromatography using AG^®^ 1-X8 resin (Bio-Rad Laboratories GmbH, Feldkirchen, Germany) was then performed to separate the ^64^Cu. Subsequently, the ^64^Cu was eluted from the column using 0.1 M HCl, and the resulting solution was evaporated in a 1.5 mL glass crimp vial.

#### 2.2.6. Production of Cu-TPP and [^64^Cu]-TPP

The complexation of copper was performed according to a procedure adapted from Fagadar-Cosma et al. [46]. In a 1.5 mL glass crimp vial, a blend of Cu(II)acetate and TPP (0.2 µmol, each) in 1 mL of ethanol underwent reflux at 85 °C for six hours using a Heidolph MR Hei-Standard hot plate (Heidolph Instruments, Kelheim, Germany) equipped with an aluminum heating block. Successful complexation was confirmed through UV/Vis spectroscopy by comparing the spectrum to the spectrum of a reference sample of commercial Cu-TPP.

The complexation of ^64^Cu was achieved via an adapted procedure. An amount of 0.4 µmol TPP in 250 µL THF was added to the dry [^64^Cu]CuCl_2_ precipitate, obtained as described above. The activity was 3.25 MBq ^64^Cu, corresponding to 356 fmol ^64^Cu. The mixture was refluxed at 75 °C for 3 h in a 1.5 mL glass crimp vial. Successful complexation was confirmed by adding 10 µL of the reaction mixture to a 250 µL:250 µL two-phase mixture of octanol and water. After 15 min of mixing, a microliter syringe was used to sample 100 µL of each phase, and the ^64^Cu activity was measured with a WIZARD 3” 1480 automatic gamma counter (Perkin Elmer, Waltham, MA, USA).

#### 2.2.7. ^64^Cu Plastics Labeling

To label plastics with ^64^Cu, 25 mg of particles (PE, PA, PVDC, PET) or 100 µL of particle dispersion (1 mg of particles, PS) was treated with 300 µL of a 1:1 (PE, PET, PS) or 1:9 (PA, PVDC) mixture of THF:DI water with 500 kBq [^64^Cu]-TPP directly added from the complexation reaction mixture. The samples were agitated for 20 min and left open overnight for the THF to evaporate. To determine radiolabeling yields, DI water was added to the samples to achieve a final volume of 1 mL. Afterward, the samples were sonicated for 2 min and centrifuged using an Eppendorf MiniSpin^®^ centrifuge (15 min, 14,100× *g*). The activity in the supernatant was checked using an automatic gamma counter. This washing procedure was performed two times. Similarly, 1 mL of DI water was added to the particle samples for radiolabel stability tests. The samples were left overnight, and the activity in the supernatant was checked the following day after centrifugation.

## 3. Results

### 3.1. System Component Characterization

#### 3.1.1. Particle Characterization

The size, morphology, and surface texture of the particles were analyzed using light microscopy and scanning electron microscopy (see Figures S4 and S5). Except for the PS size standard, the samples had broad size distributions and were slightly to highly poly-disperse (see Table 1 and Figure S6). However, the surface textures observed in SEM and EDX spectra were similar between particles of different size fractions, proving the compositional uniformity of the samples (see Figure S7). PE and PA consist of white powders containing mostly spherical to slightly oval particles with relatively narrow size distributions in the tens of micrometers range, displaying a surface texture of rounded features. PVDC consists of a pale yellow powder containing irregularly shaped particles with a very wide size distribution. Most particles were approximately 100 μm in size, with a surface texture characterized by rounded features. PET presents itself as a white powder composed of unevenly shaped fragments. These fragments vary in size, spanning hundreds of micrometers. The surface texture contains sharp edges.

#### 3.1.2. Dye Characterization

To gain a deeper understanding of the dye incorporation mechanism and labeling efficiency, we performed experiments using widely used fluorescent dyes, namely acridine orange, rhodamine 6G, rhodamine B, fluorescein, and eosin Y [48,49,50]. UV/Vis and fluorescence spectra in water were recorded, and the molar volumes of the dyes were calculated using the molar mass and density of the substances (see Table 2 and Figure S8).

**Figure 3:**
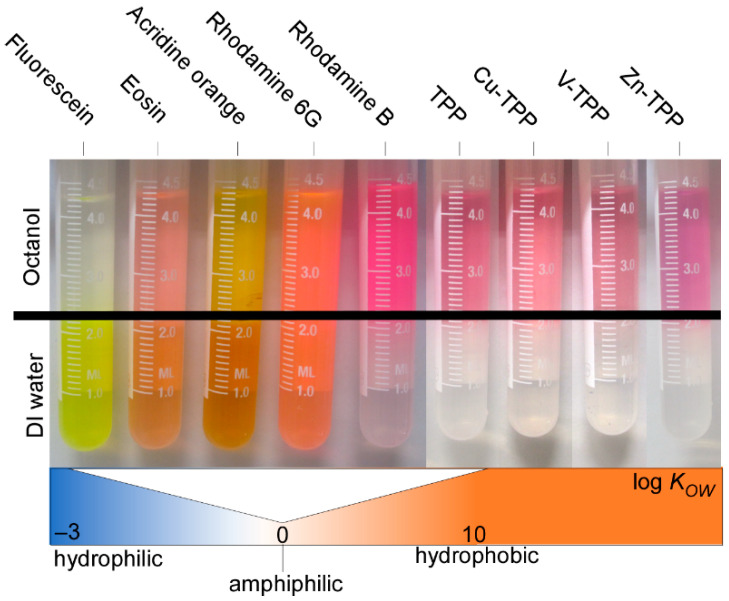
Image of octanol–water partitioning with samples ordered in sequence of increasing hydrophobicity (log *K_OW_*).

The affinity balance of the dyes interacting with the polymer matrix and the aqueous solution can be captured using the hydrophobicity of the dyes, which can be considered a major influencing factor when it comes to the efficiency of the labeling process. Hence, we first characterized the different dyes according to their hydrophobicity using the water–octanol partition coefficient *K_OW_* [51]. Values for log *K_OW_* typically ranged from −3 (very hydrophilic) to +10 (extremely lipophilic/hydrophobic). Our collection of dyes covers a range from highly hydrophilic to amphiphilic to hydrophobic compounds (see Table 2). The hydrophobicity of the dyes increased in the following order: fluorescein < eosin < acridine orange < rhodamine 6G < rhodamine B (see Figure 3 and Table 2). The TPPs utilized were entirely hydrophobic.

#### 3.1.3. Plastics Swelling Behavior

The swelling behavior of the plastics to be labeled is the second major influence on the labeling efficiency. The plastic particles should be inert in water and swell with the solvent. The solvent should be miscible with water and easily removed via gentle heating, vacuum suction, or slow evaporation. For an optimum labeling process, a reversible swelling behavior is recommended. The assessment of any expected swelling can be accomplished using the Hansen Solubility Parameters [39,52]. They probe the compatibility of the plastic matrix with a potential solvent based on the cohesion energy of the substances. Three contributions to the cohesion energy are considered: the dispersion forces, the dipolar interaction forces, and hydrogen bonding. The contributions are parameterized using three parameters, namely $\delta_{d}$, $\delta_{p}$, and $\delta_{h}$, given in units of MPa^½^ (for a detailed account, see Hansen, 2000) [39]. The more similar the three parameters are for the polymer and the solvent, the more likely the solvent will cause significant swelling, as “like dissolves like”. The similarity is evaluated using the so-called HSP distance *R_a_*, which is calculated using the Hansen parameters for the solvent and polymer according to the following formula [53,54]:

(2) $$R_{a}=\sqrt{4{\left(\delta_{d,S}-\delta_{d,P}\right)}^{2}+{\left(\delta_{p,S}-\delta_{p,P}\right)}^{2}+{\left(\delta_{h,S}-\delta_{h,P}\right)}^{2}}$$

The formula computes the distance between the interacting substances in the three-dimensional Hansen space, which is defined by the three parameters $\delta_{d}$, $\delta_{p}$ and $\delta_{h}$. The higher the HSP distance, the less likely the polymer is to dissolve/swell in the solvent. For further evaluation, a sphere of good solvents in Hansen space can be experimentally established around a center point defined by the parameters of the polymers. The sphere has the interaction radius *R*_0_ in Hansen space. Good solvents, with Hansen parameters close to the plastic in question and, consequently, low HSP distances *R_a_*, fall within the sphere. Bad solvents with high *R_a_* values lie outside of the sphere. Solvents can be classified using the Relative Energy Difference (*RED*) [55]:

(3) RED=RaR0

Good solvents, located inside of the sphere, are characterized by a *RED* < 1, while bad solvents, located outside of the sphere, are characterized by a *RED* > 1.

We can identify tetrahydrofuran and dioxane as the most promising candidates using the extensively tabulated literature HSP values for the used polymers and different common water-miscible solvents (see Table 3) [39]. *R_a_* ranges from 2.5 to 6.8 for these two solvents, while the resulting *RED* is consistently below 1, ranging from 0.45 to 0.94, for all of the polymers in question (see Table 4).

**Figure 4:**
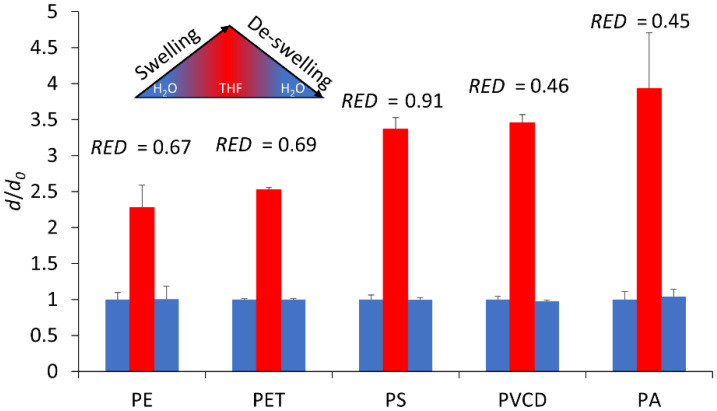
Swelling behavior of particles in 1:9 THF:DI water and de-swelling after THF removal, quantified based on the relative particle diameter (*d*/*d*_0_) in relation to the starting diameter *d*_0_, as measured via DLS before the addition of the THF (blue), after the addition of THF (red), and after the removal of the THF (blue again) (error bars = standard deviation of five consecutive DLS measurements).

Consequently, a series of swelling experiments were conducted with aqueous mixtures containing varying levels of THF (in favor of the substance of high concern 1,4 dioxane [58]), plus 2 wt% of pluronic surfactant, to ensure the dispersion of the particles [21,37]. Sampling and microscopic size evaluation at the different stages (pristine, THF-swollen, and de-swollen) showed a successful swelling for all particles. However, it was not feasible to evaluate the process’s reversibility due to the high polydispersity of most samples. Therefore, DLS measurements, singling out the nanosized particle fraction via controlled settling, were used to evaluate the swelling behavior using 1:9 THF:DI water mixtures. We observed a significant increase in particle diameter for all of our plastics, which was reversed when removing the THF (see Figure 4). No changes in the morphology or surface texture of the particles were detectable via SEM (see Figure S10). The relative increase in diameter *d/d*_0_ inversely followed the *RED* values obtained from the HSP screening, with the exception of PS. However, given that PS was the sole nanoplastic used and in a pre-made suspension, a different concentration range and surface-to-volume ratio was used in this experiment, which accounts for the discrepancy. The size increase due to swelling in THF progresses in the order of PE < PET < PS < PVDC < PA.

Further experiments with different THF concentrations revealed that increasing the THF:DI water ratio to 1:1 was possible for all particles, except for PA and PVDC. PA and PVDC exhibited irreversible swelling and the coalescence of the polymer particles into large aggregates when the THF content was raised. This finding is consistent with the HSP screening results, which identified THF as an effective solvent for these two materials, as evidenced by the lowest *RED* values.

### 3.2. Fluorescent Dye Labeling of Micro-/Nanoplastics

**Figure 5:**
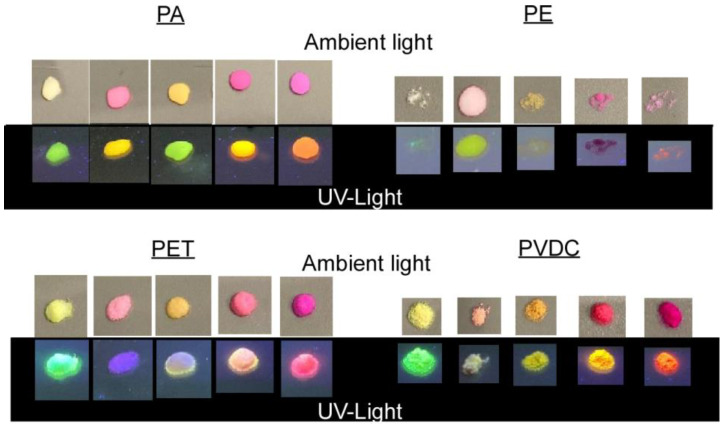
Powder samples of plastics labeled via high-dye-concentration screening tests under ambient and UV light.

For fluorescent dye labeling, plastic particles were exposed to dye dissolved in compatible THF:DI water mixtures (1:1 for PE, PET, PS; 1:9 for PA, PVDC) while shaking for 20 min. Afterward, the THF was allowed to evaporate overnight (see Figure 2). In a screening with high dye concentration, all plastics were successfully labeled using any of the dyes (see Figure 5).

**Figure 6:**
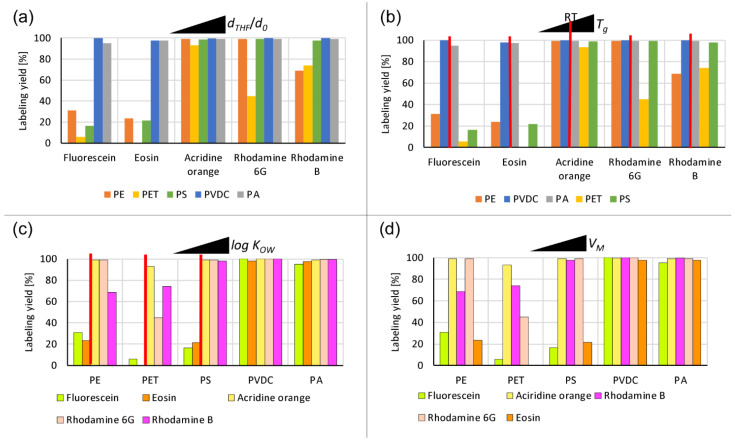
Labeling yields for fluorescent dye incorporation into plastics (measured via the UV/Vis spectroscopy of the supernatant after four washing steps): (**a**) labeling yields grouped by dye type, ordered by the increasing swelling (*d_THF_*/*d*_0_) of the plastics; (**b**) labeling yields grouped by dye type, ordered by the increasing glass transition temperature *T*_g_ of the plastics (room temperature (RT) indicated by a red line); (**c**) labeling yields grouped by particle type, ordered by the increasing hydrophobicity (log *K_OW_*) of the dye (hydrophobicity threshold indicated by a red line); (**d**) labeling yields grouped by particle type, ordered by the increasing dye molar volume *V_M_*.

Lower dye concentrations revealed differences. Firstly, it should be noted that the yield was not decreased due to the leaching of particle-incorporated dyes after four washing steps following labeling. Dyes incorporated into particles during the labeling process appeared to be stably bound. The achieved yields resulted from a complex interplay between various influencing factors. Several trends can be identified from our dataset (see Figure 6).

The particles with the largest observed size expansions during swelling and lowest *RED* values, i.e., PVDC and PA, incorporated all of the dyes with a yield close to 100% (see Figure 6a). A clear hydrophobicity threshold can be identified for the other plastics, most readily observed for PS. A notable yield increase is visible for dyes with a log *K_OW_* value equal to and above that of acridine orange (see Figure 6c). The more hydrophilic dyes, namely fluorescein and eosin, are not easily incorporated to a large degree into the less swelling particles, such as PE, PET, and PS. While hydrophobicity above the abovementioned threshold generally results in a higher labeling yield, the trends observed for increasing hydrophobicity do not align with the measured log *K_OW_* values. This result implies that other mechanisms play a role in the incorporation process. One of these mechanisms is indicated by the overall good performance of acridine orange. Labeling yields with acridine orange of nearly 100% are achieved for all plastics tested (see Figure 6a). Acridine orange is the only dye used that does not belong to the triarylmethine dye family (see Figure S2). The tricyclic acridine-based structural motif of acridine orange, formed by conjugated heteroaromatics, allows easy intercalation due to its flattened shape [59,60]. Among the dyes utilized, it has the smallest size in terms molar volume, except for the hydrophilic fluorescein (see Table 2). Ordering the labeling yields in the sequence of increasing molar volume of the dyes shows a trend of generally decreasing yields with the increasing molar volume of the respective dye. The notable exception to this trend is fluorescein, the hydrophilicity of which prohibits high-yield labeling for most plastics, despite its relatively small molar volume. Only the highly swelling PVDC and PA incorporate it to a large degree. Excluding the fluorescein exception, the effect of the molar volume is most easily observed in the PET series. A gradual decline in yields can be seen with an increase in the dye’s molar volume (see Figure 6d). PE exhibits a similar pattern with a discrepancy in the arrangement of the two rhodamines. For the highly swelling PVDC and PA, only a subtle yield decrease can be identified at the highest molar volume (eosin). A similar but more pronounced yield drop is visible for PS.

Ordering the data by increasing glass transition temperature of the plastics (see Table 1) reveals no apparent trend in yields for plastics with glass transition temperatures either below or above the experimental temperature (see Figure 6b). Other factors potentially affecting the final yields are the particle surface area-to-volume ratio and the particle number concentration, which could not be kept constant throughout the experimental series due to the polydispersity of our samples.

From the data, three conclusions can be drawn about the labeling process efficiency:

1. The labeling yield increases with more efficient swelling behavior;
2. The labeling yield increases with dye hydrophobicity, with a tentative threshold identifiable for slightly hydrophilic to amphiphilic log *K_OW_* values close to 0;
3. The labeling yield decreases with dye molar volume, with possible shape effects for flat molecular structures.

**Figure 7:**
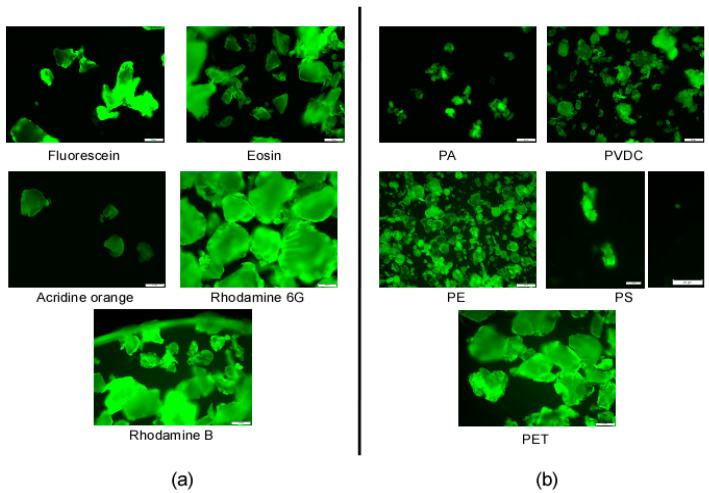
Fluorescence microscopy images of labeled plastics: (**a**) images of labeled PET particles using all of the different dyes; (**b**) images of all particle types labeled with rhodamine B. PS images for aggregate and single particles; (scale bar = 50 µm (PA, PE, PET, PVDC); scale bar = 10 µm (PS)).

All labeled particles, including those with low yields based on UV/Vis spectroscopy measurements, are easily identifiable through fluorescence microscopy (see Figure 7).

### 3.3. Radiolabeling of Micro-/Nanoplastics

The potential radiolabeling of plastics using the same swelling and in-diffusion mechanism utilizes complexed radiometals. Based on the results described above, we used the hydrophobic porphyrin derivative TPP as a radiometal carrier. It exhibits a relatively flat porphyrin structure (see Figure S2) and is known to form stable complexes with various metal ions [61,62]. We use cyclotron-produced ^64^Cu to introduce a radiolabel into our plastic samples [42].

#### 3.3.1. ^64^Cu Complexation

The initial step toward successful radiolabeling involves complexing the radiometal with the intended carrier molecule. To synthesize metal-derivatized porphyrins, several approaches are available. They share, as a common feature, the presence of an excess amount of metal in a suitable solvent and the use of reflux at high temperatures, leading to the quantitative metalation of porphyrin molecules. To test the complexation of copper by the TPP, we first performed an experiment with a 1:1 TPP:copper (as acetate) mixture in ethanol following procedures in the literature [46]. Refluxing the mixture of Cu^2+^, TPP, and ethanol for 6 h resulted in quantitative porphyrin metalation, confirmed via a band shift in the UV/Vis spectrum (see Figure S11).

**Figure 8:**
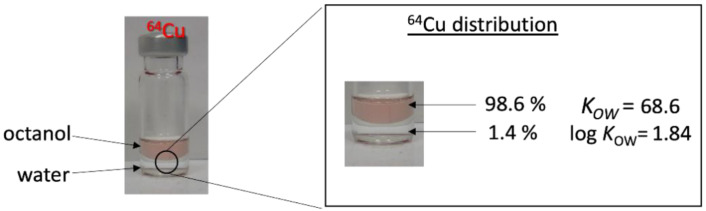
Complexation of radiocopper ^64^Cu by TPP, proven via octanol–water partitioning.

In the case of the radiolabel, the amount of porphyrin will always exceed the amount of radiometal ion (provided that there is no non-radioactive carrier present). For example, the 3.25 MBq of ^64^Cu used in our experiment corresponds to 356 fmol of substance. Thus, a quantitative complexation can be expected, even at lower temperatures. To avoid time-consuming isolation and purification procedures, particularly given the half-life of ^64^Cu, our approach was to use TPP to complex radiocopper in THF at reflux (boiling point: 64 °C). This approach has the potential for a one-pot radiolabeling methodology, which involves a complexation step in THF, followed by the addition of plastics dispersed in water, without the need for the separation or purification of the [^64^Cu]-TPP. Consequently, the complexation procedure was performed with 3.25 MBq of cyclotron-produced ^64^Cu, now in massive undersupply of about 1:10^6^, ^64^Cu:TPP. Due to the low concentration, it was impossible to detect complexation through UV/Vis spectroscopy. Instead, complexation was tested through the log *K_OW_* of the system after 3 h of reflux. As the TPP quantitatively accumulates in the octanol phase, any complexed radiometal is also transferred to the hydrophobic phase. We observed an accumulation of 98.6% radiocopper in the octanol phase, indicating the successful near-quantitative hydrophobization of the copper, ready for use in the in-diffusion radiolabeling of plastics (see Figure 8).

#### 3.3.2. ^64^Cu Radiolabeling

As a first test, incorporating TPP-complexed metals into plastics was non-radioactively attempted using commercially available metalo-TPPs. Specifically, we used copper, vanadate, and zinc TPPs, reflecting the potential radiolabels ^64^Cu, ^48^V, and ^65^Zn. Employing the same procedure used for fluorescent dye labeling, we achieved yields very close to 100% for all plastics tested (see Figure S12). The radiolabeling was performed using the [^64^Cu]-TPP dissolved in THF, prepared as described above, and directly taken from the complexation reaction mixture without further processing. As with the fluorescent dyes, a short agitation period was followed by allowing the THF to evaporate overnight. This resulted in radiolabeled micro-/nanoplastics with radiolabeling yields at about 90% (see Table 5). The labeled particles had a specific activity of approximately 15 kBq/mg for PE, PA, PET, and PVDC and 400 kBq/mg for PS (as the nano-PS size standard was obtained in colloidal dispersion, 25 times less particle mass was used in this experiment). In particle fate studies using the labeled plastics, particle masses can be calculated via a simple activity measurement using the specific activities of the particles set by the labeling process. No extensive sample preparation is required, since the radiation can be effortlessly detected outside of the sample. This process allows the detection of tens of nanograms or even single nanograms (see Table 5). These values are not to be understood as fundamental limits, but could easily be improved using higher ^64^Cu activities in the labeling procedure.

**Figure 9:**
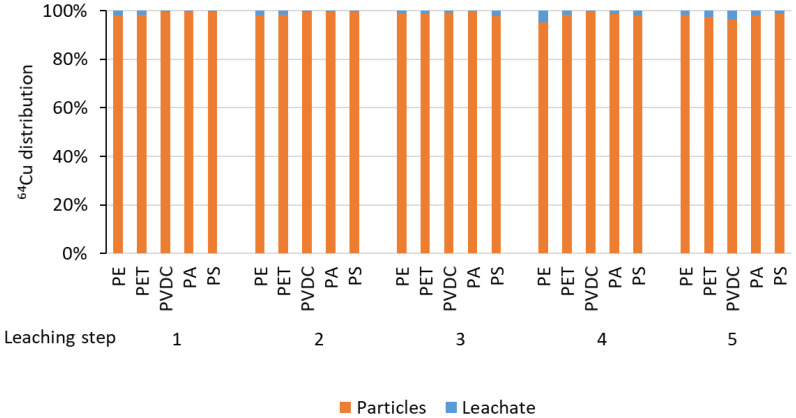
^64^Cu distribution among particle samples and supernatant leaching solution for five leaching steps.

To test the stability of the radiolabel, leaching experiments were performed, spanning five steps within one week, covering the decay time of the ^64^Cu (conventionally, the maximum duration of radiotracer experiments is considered to be up to 10 half-lives, *t_1/2_*(^64^Cu) = 12.7 h). Any leaching observed during this period did not exceed single-digit percentages, falling within the error margin caused by the imperfect separation of particles from the supernatant solution (see Figure 9).

## 4. Discussion

Our results demonstrate the usefulness of a HSP and hydrophobicity screening in selecting the solvent and label carrier. PA and PVDC exhibited the most pronounced swelling of the plastics in accordance with the HSP screening. These polymers consequently quantitatively incorporated all of the dyes, highlighting the swelling mechanism’s predominant importance. A HSP screening for other common plastics, including polypropylene (PP), polyvinyl chloride (PVC), polybutadiene (PB), polymethylmethacrylate (PMMA), polyvinyl alcohol (PVA), and polycarbonate (PC), indicates the potential for labeling a wide range of plastic materials with a THF-based procedure (*RED* < 1, Table S1). For cases like polyacrylonitrile (PAN, *RED* = 1.19, see Table S1), a HSP screening can aid in selecting an alternative solvent. Hydrophobicity, as quantified by the log *K_OW_*, proved to be a valuable guide but does not capture every aspect of the system. It may be beneficial to extend the HSP screening to the dye in interaction with the solvent and/or polymer matrix [54,63,64]. For instance, a screening for rhodamine B demonstrates that there are low HSP distances between rhodamine B, the plastics and THF, whereas the interactions with octanol are less favorable (see Table 6). Therefore, a HSP screening for the dyes could more precisely capture the required interaction profiles than the octanol–water partition coefficient.

Discrepancies in the observed trends may arise due to variation in the speciation of the dyes caused by fluctuations in pH, which can impact hydrophobicity [65]. For instance, fully protonated fluorescein exhibits low solubility in water. Literature values for rhodamine 6G and rhodamine B indicate different hydrophobicities to those measured in this study [56]. As a result, the rhodamines might trade positions in the hydrophobicity sequence depending on minor fluctuations in reaction conditions. Deprotonation, dimer formation, and tautomerization can cause changes in hydrophobicity and effective molecular size, which may hinder or facilitate the labeling process [66,67,68]. In any case, dye-dependent optimized procedures may improve labeling yields [69,70]. We observed no impact of the glass transition temperature of the plastics and no evidence of dye leaching from the particles. Furthermore, our experimental results indicate that the yields for rhodamine B incorporation do not align with the *R_a_* values acquired from an HSP screening of interactions between rhodamine B and the plastics (see Table 6). Therefore, we may conclude that for our plastics and experimental conditions, the actual chemistry of the polymer material only plays a minor role. It seems limited to providing a suitable matrix to swell with the THF. The solvent effectively eliminates the physical obstacle to molecular diffusion into a rigid polymer below the glass transition temperature [71]. The solvent swelling notably lowers the glass transition temperature and expands the polymer mesh, only leaving an inhibiting effect on diffusion for the largest dye molecules [38,72,73]. Mesh size, crosslinking [74], the entanglement of polymer strands [75], and the semi-crystallinity of the plastic matrix, as well as polymer particle size [76], control this factor on the side of the polymer [77,78]. On the side of the dye, the actual effective size can vary with speciation and is influenced by the anisometric shape of flat molecular structures [79].

The porphyrin structure allowed the successful quantitative complexation and hydrophobization of radiocopper ^64^Cu, thus allowing the integration of the radiolabel into micro- and nanoplastics. As a result, the sensitive tracking of nanograms of particles is enabled, as the radiolabel is stably bound inside of the polymer matrix. Furthermore, autoradiography and positron emission tomography techniques can achieve 2D spatial distribution data and 4D spatiotemporal tracking, respectively.

Porphyrins can complex various di- and trivalent metals, prominently evidenced by the natural metalo-porphyrin derivatives hemoglobin (Fe) and chlorophyll (Mg). This has the potential to introduce a wide range of radiotracers into plastic systems. Adaptations to the complexation procedure may be required to achieve efficient hydrophobization through TPP metalation when using other radiometals [61,62]. Depending on the propensity of the metal to form porphyrin complexes, it may be necessary to increase the reaction temperature and/or use a different solvent. As a result, an additional step in the procedure would be required to separate the metal-TPP. However, hydrothermal and solvent-free metalations offer a solution to this issue [80,81]. The derivatization of the porphyrin structures may allow the tailoring of the carrier–polymer interactions to specific systems to enhance the yields and stability. Potential radiotracer candidates have a broad range of half-lives, ranging from hours to days to years, offering numerous tracing applications (see Table 7).

## 5. Conclusions

The radiolabeling of the common micro/nanoplastics PET, PS, PA, PE, and PVDC was successfully achieved through an in-diffusion technique with [^64^Cu]-TPP in THF. The study demonstrated the practicality of utilizing HSP and log *K_OW_* screenings for selecting suitable experimental conditions and radiometal carrier systems. An efficient labeling outcome was found to be influenced by the swelling behavior of the polymer and the molecular size and hydrophobicity of the label. The established experimental procedure allows facile one-pot radiolabeling of polymer particles, enabling sensitive and selective detection, regardless of the elemental and particle backgrounds. The technique is versatile and can be applied to various tracer and polymer particles types, with HSP calculations serving as a valuable guide for adjustments to the procedure.

## Figures and Tables

**Table 1:** Plastic particle sizes measured via light microscopy and DLS (see also Figure S6) and polymer glass transition temperatures *T_g_* from [47].

Material	Median Size [µm]	Average Size [µm]	Std. Dev. [µm]	Std. Dev. [%]	*T_g_* [°C]
PE PS PET PVDC PA	42.58 0.1078 197.4 84.10 31.07	41.96 0.1078 208.7 91.97 32.72	14.95 0.0003 89.76 63.18 6.99	35.6 0.3 43.0 68.7 21.4	−130 … −100 80 … 105 70 … 85 −18 … 15 40 … 50

**Table 2:** Structural data, UV/Vis/fluorescence spectral data (n.d. = not determined), and octanol–water partition coefficients of fluorescent dyes used.

Dye	Molar Mass [g/mol]	Density [g/mL]	Molar Volume [mL/mol]	Peak Maximum *λ_A_*/*λ_E_* [nm]	Extinction Coefficient [L/molcm]	log *K_OW_*
acridine orange rhodamine 6G rhodamine B fluorescein eosin TPP	308.81 479.02 479.02 376.28 698.86 614.74	1.001 1.260 1.310 1.601 1.018 1.270	308.5 380.2 365.7 235.0 685.5 485.0	492/530 526/550 555/574 489/514 516/538 440/n.d.	17753 83476 89191 33148 51347 n.d.	−0.38 0 1.6 <−3 * −0.92 >10 *

* No measurable dye absorbance in the water or octanol phase.

**Table 3:** Hansen Solubility Parameters * in MPa^½^ for plastics and common water-miscible solvents, as well as for octanol and rhodamine B.

Material	$\delta_{d}$	$\delta_{p}$	$\delta_{h}$	*R*_0_
PE PS PET PVDC PA	17.67 19.46 18.43 18.12 17.5	5.27 6.30 6.30 8.08 5.3	3.25 4.12 7.30 5.66 10.1	7.63 7.24 4.93 9.56 5.5
THF water acetone ethanol DMF DMSO 1-propanol acetonitrile 1,4-dioxane methanol isopropanol acetic acid	16.8 15.5 18.4 15.8 17.4 18.4 16 15.3 19 15.1 15.8 14.5	5.7 16 10.4 8.8 13.7 16.4 6.8 18 1.8 12.3 6.1 8	8 42.3 7 19.4 11.3 10.2 17.4 6.1 7.4 22.3 16.4 13.5	- - - - - - - - - - - -
1-octanol rhodamine B	17 17.8	3.3 4.3	11.9 6.2	- -

* Tabulated values from [39] for plastics (see Figure S9) and solvents, as well as from [56] for rhodamine B; common water-miscible solvents according to [57].

**Table 4:** HSP distance *R_a_* [MPa^½^]/*RED* of plastics for different common water-miscible solvents and octanol. *RED* < 1 defines good solvents, as indicated in bold/underlined.

Material	PE	PS	PET	PVDC	PA
THF water acetone ethanol DMF DMSO 1-propanol acetonitrile 1,4-dioxane methanol isopropanol acetic acid 1-octanol	5.08/**0.67** 40.73/5.34 6.52/**0.85** 16.95/2.22 11.67/1.53 13.20/1.73 14.62/1.92 13.88/1.82 6.03/**0.79 ** 20.95/2.75 13.70/1.80 12.36/1.62 8.98/1.18	6.61/**0.91** 40.18/5.55 5.44/**0.75** 17.12/2.36 11.10/1.53 11.98/1.65 14.98/2.07 14.49/2.00 5.64/**0.78 ** 21.03/2.91 14.29/1.97 13.75/1.90 9.68/1.34	3.39/**0.69** 36.79/7.46 4.11/**0.83** 13.43/2.72 8.66/1.76 10.51/2.13 11.22/2.27 13.33/2.70 4.64/**0.94 ** 17.48/3.54 10.52/2.13 10.16/1.35 6.19/1.26	2.56/**0.46** 34.17/6.21 6.23/1.13 10.50/1.91 8.49/1.54 11.25/2.04 8.03/1.46 14.02/2.55 5.34/**0.97 ** 14.46/2.70 7.20/1.31 7.41/1.35 2.87/**0.52**	4.26/**0.45** 37.85/3.96 2.74/**0.29** 14.52/1.52 8.09/**0.85** 9.49/**0.99** 12.55/1.31 11.42/1.19 6.75/**0.71 ** 18.20/1.9 11.87/1.24 10.67/1.12 8.17/1.03

**Table 5:** Radiolabeling yields and detection limits.

Material	Radiolabeling Yield [%]	Specific Activity [kBq/mg]	Detection Limit ^1^ [ng/µg/L]
[^64^Cu]TPP [^64^Cu]PE [^64^Cu]PS [^64^Cu]PA [^64^Cu]PET [^64^Cu]PVDC	98.6 87.7 92.4 97.1 88.4 93.2	12760 14.5 409.9 17.5 16.2 15.9	0.078/0.004 69/3.4 3/0.1 58/2.9 62/3.1 67/3.3

^1^ Detection using a Perkin Elmer WIZARD 3” 1480 automatic gamma counter, assuming a detection limit of 1 Bq and a maximum sample size of 20 mL.

**Table 6:** HSP distance *R_a_* [MPa^½^] for rhodamine B in combination with different solvents and polymers.

Material	*R_a_*
THF water 1-octanol PE PS PET PVDC PA	3.03 38.23 6.00 3.12 4.39 2.61 4.07 3.87

**Table 7:** Potential radiotracers for porphyrin-labeling strategies *.

Metal	Radiotracer	*t_1/2_*
Cu Zn Mn Co Fe Ni Pd Ag Au Ba V Cr Cd Ti P Ga In Pb Zr	^64^Cu ^65^Zn ^54^Mn ^57^Co ^55^Fe/^59^Fe ^63^Ni ^103^Pd ^110m^Ag/^105^Ag ^198^Au ^133^Ba ^48^V ^51^Cr ^109^Cd ^44^Ti/^45^Ti ^32^P ^67^Ga ^111^In ^203^Pb ^88^Zr/^89^Zr	12.7 h 244.06 days 312.3 days 271.79 days 2.737 years/44.495 days 100.1 years 16.991 days 249.79 days/41.29 days 2.69 days 10.5 years 15.9735 days 27.7025 days 462.6 days 49 years/184.8 min 14.26 days 3.2612 days 2.8047 days 59.1 h 83.4 days/78.41 h

* Based on common radiotracers according to IAEA [82,83] and the commercial availability of M-TPP derivatives [84,85,86], indicating the feasibility of synthesis and stability of the product.

## Data Availability

The data presented in this study are openly available in RODARE at http://doi.org/10.14278/rodare.2476 (accessed on 20 September 2023) [87].

## Supplementary Materials

The following supporting information can be downloaded via this link: https://www.mdpi.com/article/10.3390/nano13192687/s1, Figure S1: Chemical structures of plastics used; Figure S2: Chemical structures of dyes/carriers used; Figure S3: Viscosity and refractive index of THF:water mixtures; Figure S4: light microscopy images of microplastics; Figure S5: SEM images of plastic particles. Figure S6: Size distributions of plastic particles; Figure S7: EDX spectra of microplastics; Figure S8: UV/Vis and fluorescence spectra of dyes; Figure S9: Box-plot of HSPs of plastics; Figure S10: SEM images of particle before and after swelling test; Figure S11: UV/Vis spectral shift of Cu-TPP; Figure S12: Yields for metalo-TPP labeling of plastics; Table S1: HSP screening of additional plastics in interaction with THF.
