# Supplementary material for: Radiolabeling of Micro-/Nanoplastics via In-Diffusion
Source: Nanomaterials (Basel). 2023 Sep 30;13(19):2687. doi: 10.3390/nano13192687 (PMC10574329; doi:10.3390/nano13192687)
Supplement: Supplementary file 1 [file nanomaterials-13-02687-s001.zip › nanomaterials-2633044-supplementary.pdf]

Supporting information

for

Radiolabeling of Micro-/Nanoplastics via In-diffusion

Alexandra Stricker [1], Stephan Hilpmann[2], Alexander Mansel[1], Karsten Franke[1] and Stefan Schymura[1*]

[1] Helmholtz-Zentrum Dresden-Rossendorf, Institute of Resource Ecology, Research Site Leipzig, Permoserstr. 15, 04318 Leipzig, Germany;
[2] Helmholtz-Zentrum Dresden-Rossendorf, Institute of Resource Ecology, Bautzner Landstraße 400, 03148 Dresden, Germany;

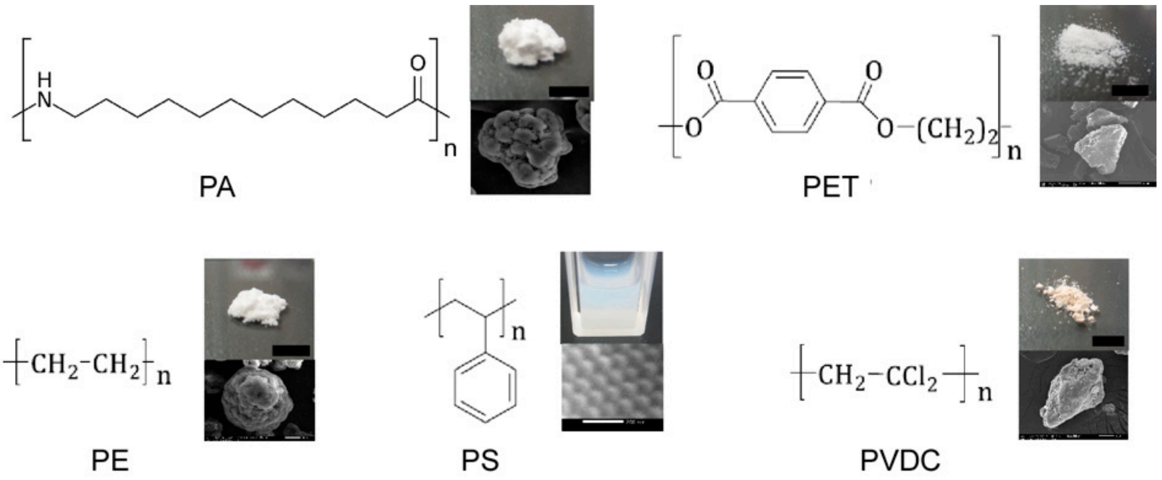

Figure S1: Plastics chemical structure, appearance of particle powders (scale bar: 2 mm), and electron microscopy of single particles (see Figure S5 for larger SEM images).

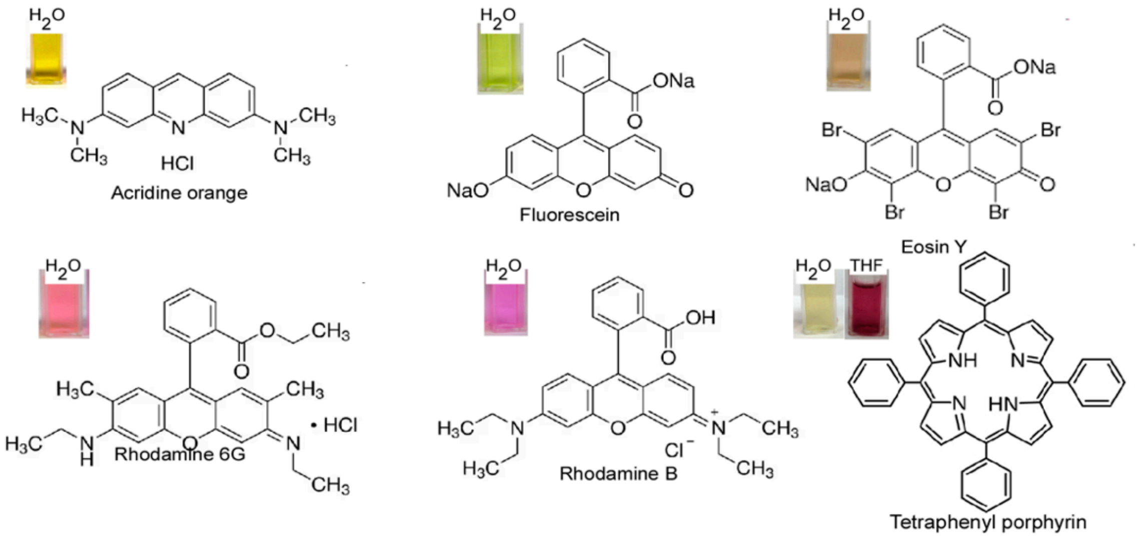

Figure S2: Chemical structures of dyes and photographs of solutions under ambient light.

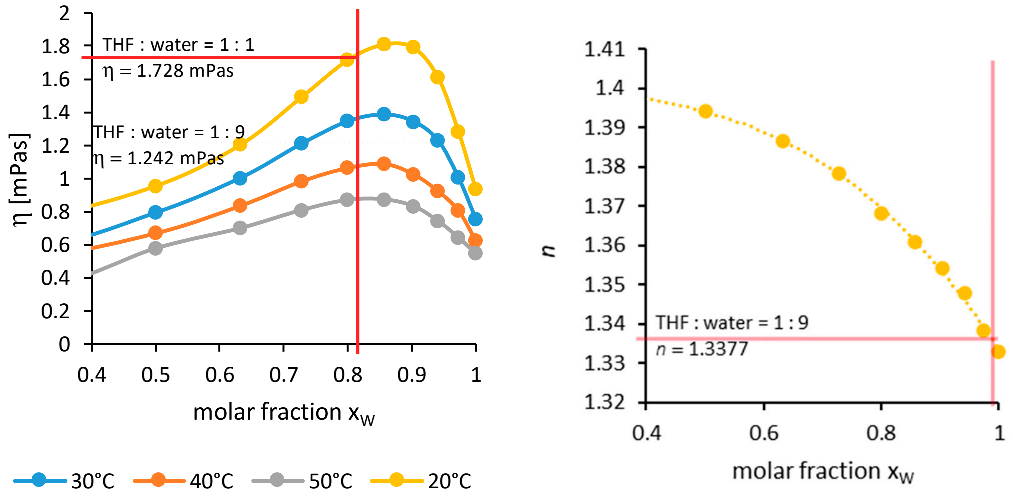

Figure S3: Viscosity and refractive index of aqueous THF mixtures, according to Nayak et al. 2004.

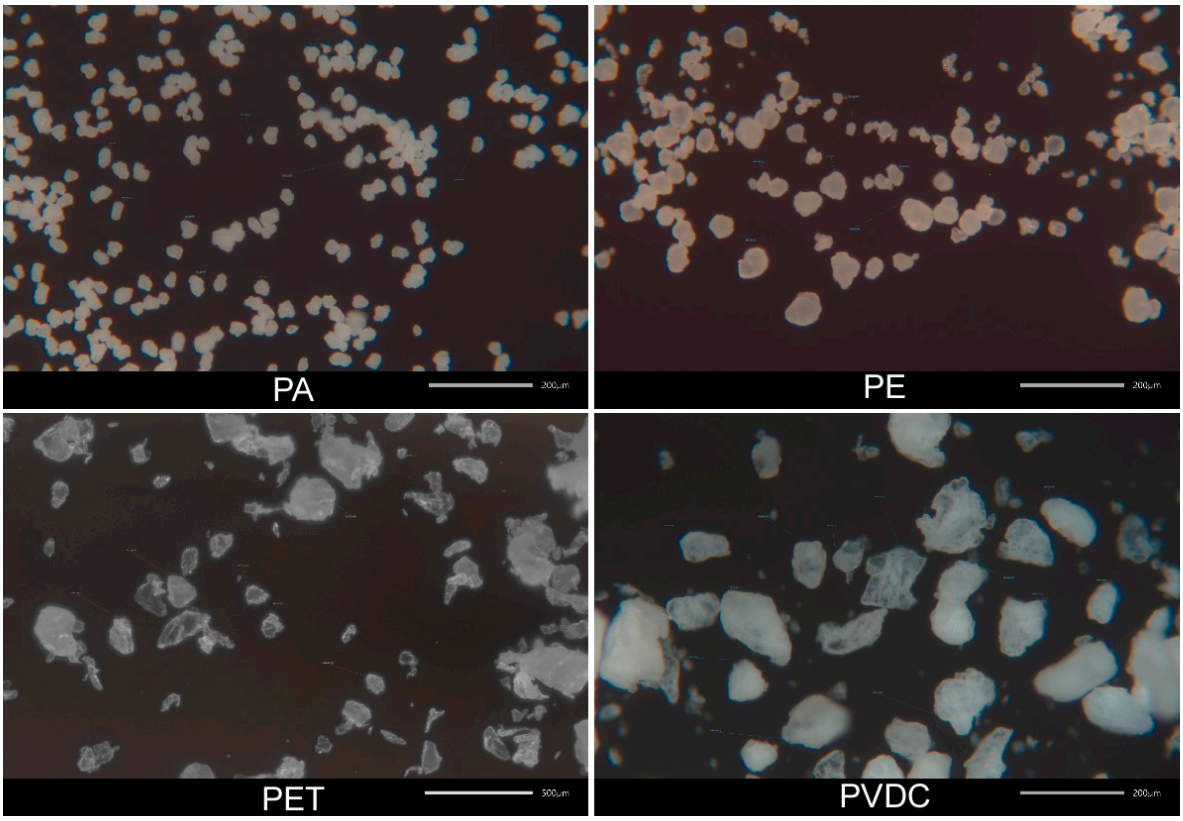

Figure S4: Light microscopy images of microplastic particles.

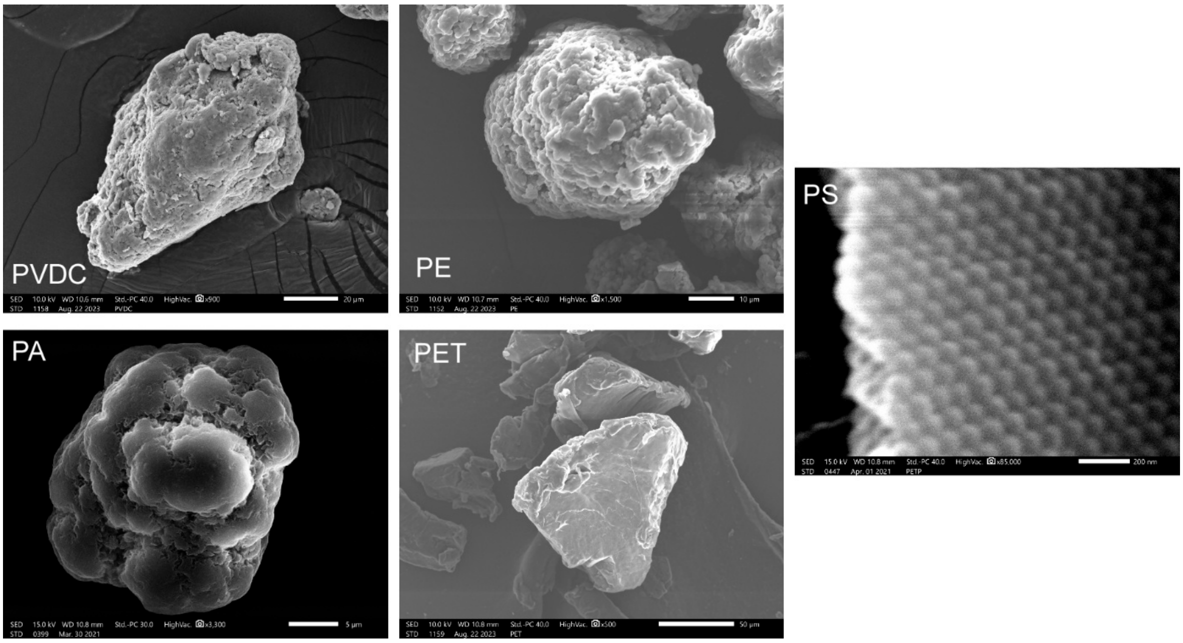

Figure S5: Scanning electron microscopy images of plastic particles.

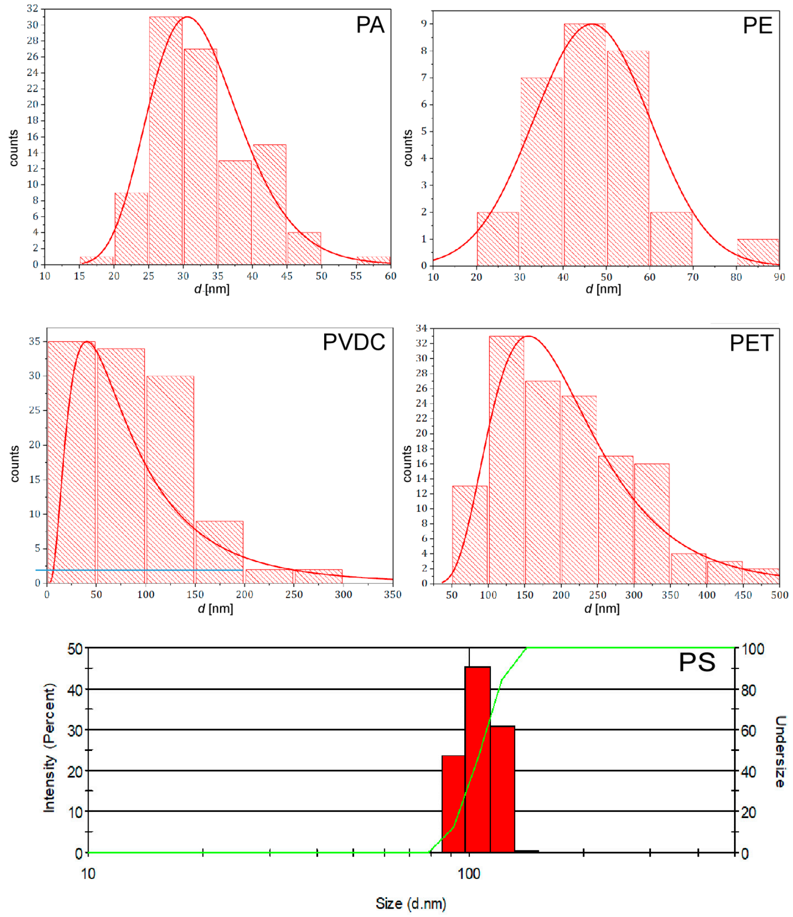

Figure S6: Size distributions of microplastic samples obtained from light microscopy images (PA (n= 118), PE (n=29), PVDC (n=112), PET (n=142)), and dynamic light scattering (PS).

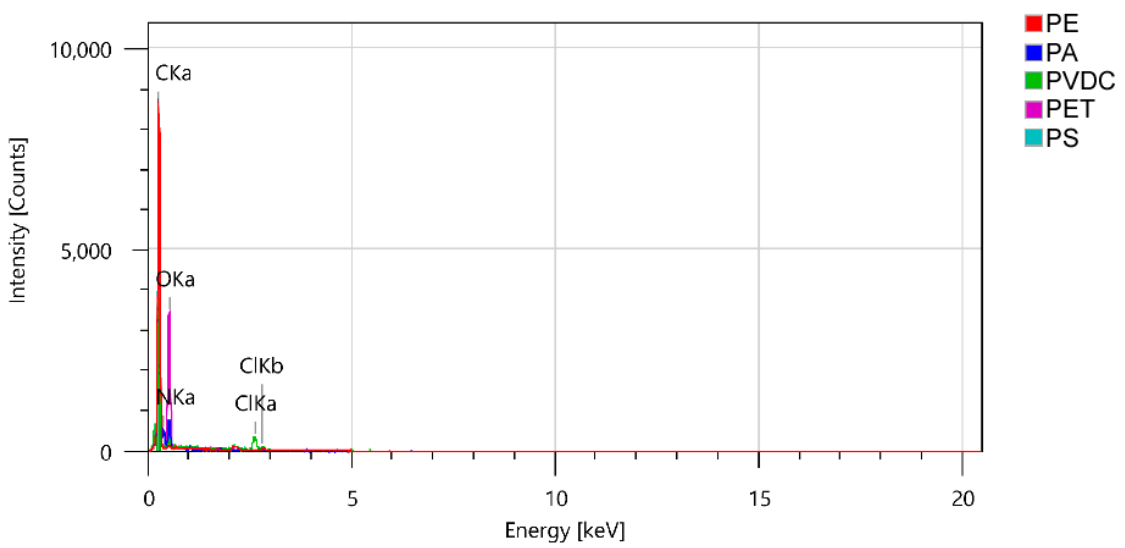

Name	C	N	O	Cl	Total
PE	100.00				100.00
PA	80.76	8.67	10.57		100.00
PVDC	81.33			18.67	100.00
PET	64.29		35.71		100.00
PS	91.86		8.14		100.00

Atom%

Figure S7: EDX spectra and element atom percentages for microplastics.

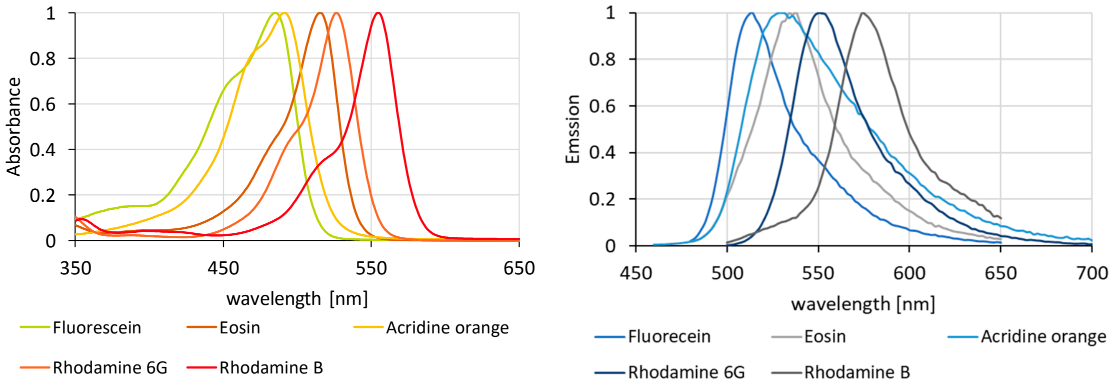

Figure S8: (left) UV/Vis spectra of fluorescent dyes; (right) fluorescence emission spectra of fluorescent dyes.

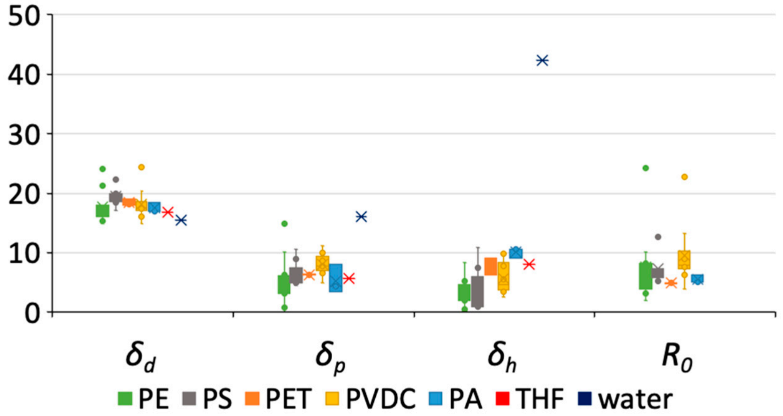

Figure S9: Box-plot of Hansen Solubility Parameters of plastics, and THF and water for comparison. Since the values can vary, the average of the tabulated values reported in Hansen 2000 for every material type was used for the HSP screening.

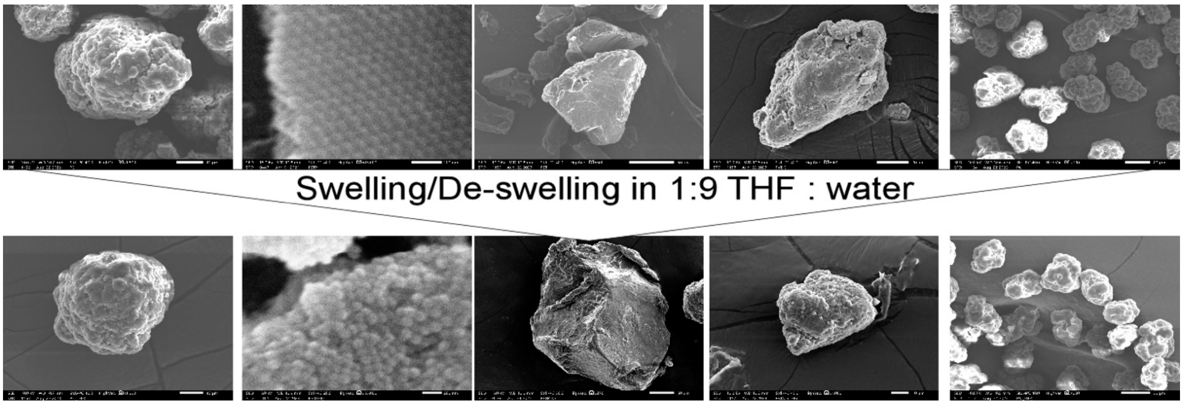

Figure S10: TEM images of pristine plastics and plastics after undergoing a swelling/de-swelling cycle with 1:9 THF:water.

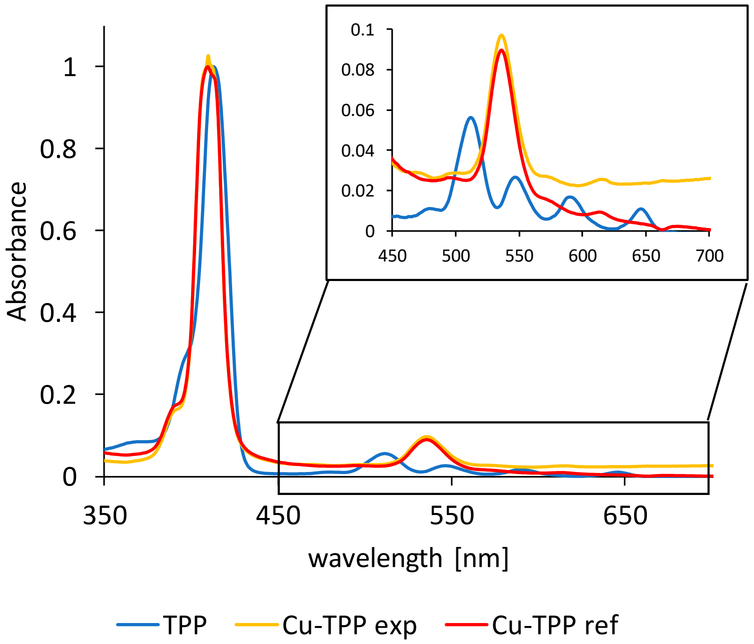

Figure S11: UV/Vis spectral shift of TPP due to metalation with Cu(II); UV/Vis spectra of pristine TPP (blue), Cu-TPP exp, Cu-TPP produced in this study (yellow), and Cu-TPP ref, commercial Cu-TPP reference sample (red). All spectra were measured in THF.

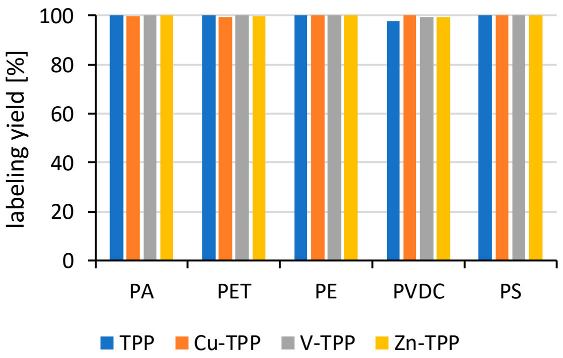

Figure S12: Labeling yields for metalo-TPP labeling of plastics.

Table S1: Hansen solubility parameters* in MPa½ and derived R_a and *RED* values for additional common plastics (polypropylene (PP), polybutadiene (PB), polyvinylchloride (PVC), polymethylmethacrylate (PMMA), polyacrylonitrile (PAN), polyvinylalcohol (PVA), polycarbonate (PC)) in interaction with THF.

**Material**	δ_d	δ_p	δ_h	R_0	R_a	*RED*
PP	18.00	3.00	3.00	8.00	6.17	0.77
PB	17.50	2.30	3.40	6.50	5.89	0.91
PVC	18.25	7.73	5.75	13.35	4.19	0.31
PMMA	17.92	10.07	4.30	9.20	6.14	0.67
PAN	21.70	14.10	9.10	10.90	12.95	1.19
PVA	14.57	13.07	10.60	10.60	9.00	0.85
PC	19.10	10.90	5.10	12.10	7.52	0.62

* Tabulated HSP values for plastics from Hansen, 2000.
